# Computational Insights into the Interactions between Calmodulin and the c/nSH2 Domains of p85α Regulatory Subunit of PI3Kα: Implication for PI3Kα Activation by Calmodulin

**DOI:** 10.3390/ijms19010151

**Published:** 2018-01-04

**Authors:** Duan Ni, Dingyu Liu, Jian Zhang, Shaoyong Lu

**Affiliations:** Department of Pathophysiology, Key Laboratory of Cell Differentiation and Apoptosis of Chinese Ministry of Education, School of Medicine, Shanghai Jiao Tong University, Shanghai 200025, China; niduan11@sjtu.edu.cn (D.N.); sjdldy@sjtu.edu.cn (D.L.); methane02@163.com (J.Z.)

**Keywords:** calmodulin, PI3Kα, K-Ras4B, molecular dynamics simulations, molecular modeling, adenocarcinoma, protein-protein interactions

## Abstract

Calmodulin (CaM) and phosphatidylinositide-3 kinase (PI3Kα) are well known for their multiple roles in a series of intracellular signaling pathways and in the progression of several human cancers. Crosstalk between CaM and PI3Kα has been an area of intensive research. Recent experiments have shown that in adenocarcinoma, K-Ras4B is involved in the CaM-PI3Kα crosstalk. Based on experimental results, we have recently put forward a hypothesis that the coordination of CaM and PI3Kα with K-Ras4B forms a CaM-PI3Kα-K-Ras4B ternary complex, which leads to the formation of pancreatic ductal adenocarcinoma. However, the mechanism for the CaM-PI3Kα crosstalk is unresolved. Based on molecular modeling and molecular dynamics simulations, here we explored the potential interactions between CaM and the c/nSH2 domains of p85α subunit of PI3Kα. We demonstrated that CaM can interact with the c/nSH2 domains and the interaction details were unraveled. Moreover, the possible modes for the CaM-cSH2 and CaM-nSH2 interactions were uncovered and we used them to construct a complete CaM-PI3Kα complex model. The structural model of CaM-PI3Kα interaction not only offers a support for our previous ternary complex hypothesis, but also is useful for drug design targeted at CaM-PI3Kα protein-protein interactions.

## 1. Introduction

K-Ras4B is situated at the crossroad of several cellular signaling pathways, and it is involved in cell growth and proliferation [1,2,3,4,5,6]. Mutations of K-Ras4B frequently occur in various kinds of cancers [3,7,8,9,10,11], and one of the most deadly of them is pancreatic ductal adenocarcinoma (PDAC) [9,12,13,14]. PDAC is known for its poor prognosis and high mortality and few patients can survive for 5 years after operations [15]. Mutation rate of K-Ras4B in PDAC can be as high as 95% [5,16,17] and accumulating evidence suggests that both calmodulin (CaM) and phosphatidylinositide-3 kinase (PI3Kα) play key roles in the K-Ras4B-driven adenocarcinoma [18,19,20,21]. 

CaM is a calcium-binding protein [22,23,24] that consists of a pair of symmetric N-lobe and C-lobe and the connecting linker [25]. Since the linker is relatively flexible, CaM can adopt two conformational topologies, the extended and the collapsed shapes. In response to calcium binding, CaM can interact with different targets [26] and it is thus involved in several intracellular signaling pathways [22,23,24,27,28,29,30]. One of the major targets of CaM is K-Ras4B [18,19,20,27]. In adenocarcinoma, GTP-bound activated K-Ras4B is specifically regulated by CaM [19,20,24], and such a finding is supported by the clinical observation that higher calcium concentration in serum can lead to the poor prognosis of the K-Ras4B-driven PDAC [23]. Thus, it is widely accepted that CaM can modulate the progression of K-Ras4B-driven cancer and one potential downstream target is the K-Ras4B-PI3K-Akt pathway [19,21,24,28]. 

PI3Kα is a downstream target of K-Ras4B [31,32] and PI3Kα-Akt signaling pathway is one of the key components in the K-Ras4B-driven tumorigenesis [21,32,33,34]. PI3Kα is a lipid kinase composed of p110α catalytic and p85α regulatory domains [35,36,37,38]. Upon activation by the phosphorylated epidermal growth factor receptor (pEGFR) and K-Ras4B, PI3Kα catalyzes the synthesis of the second messenger phosphatidylinositol-3,4,5-triphosphate (PIP_3_) and further activates the following Akt protein [34,39,40,41,42,43,44,45]. PI3Kα and mitogen-activated protein kinase (MAPK) signaling are both mediated by K-Ras4B. However, Saur et al. have recently revealed that in K-Ras4B-driven adenocarcinoma, PI3Kα signaling is abnormally stimulated by the mutated K-Ras4B while the MAPK pathway cannot be activated [21]. Hence, both CaM and PI3Kα are regarded as important regulators in the K-Ras4B-driven adenocarcinoma. Additionally, previous research has shown that CaM can directly interact with PI3Kα through its p85α subunit and enhance its activity [46]. Thus, based on these results, we have recently put forward a ternary complex model in which the coordination of CaM and PI3Kα with K-Ras4B forms a CaM-PI3Kα-K-Ras4B ternary complex, which leads to PDAC [47,48,49]. According to this hypothesis, CaM-PI3Kα-K-Ras4B interact with each other, which explains the above observation that both CaM and PI3Kα modulate the K-Ras4B-driven carcinogenesis. However, the detailed interactions between CaM and PI3Kα are still unknown.

Here, we explored the two regulatory components of the ternary complex, CaM and PI3Kα. We employed the molecular modeling and molecular dynamics (MD) simulations to explore the interactions between CaM and both the cSH2 and nSH2 domains of p85α regulatory subunit of PI3Kα. We first demonstrated that CaM can replace pEGFR to bind to the cSH2 and nSH2 domains. The potential models for the CaM-cSH2 and CaM-nSH2 interactions were unmasked. Detailed interactions of the CaM-cSH2 and CaM-nSH2 complexes were further revealed, which was applied to construct a complete CaM-PI3Kα complex. The obtained results not only offer a support for our previous ternary complex hypothesis but also provide a structural basis for future drug design targeting CaM-PI3Kα protein-protein interactions.

## 2. Results 

### 2.1. Overview of the Complex Structures

**Figure 1:**
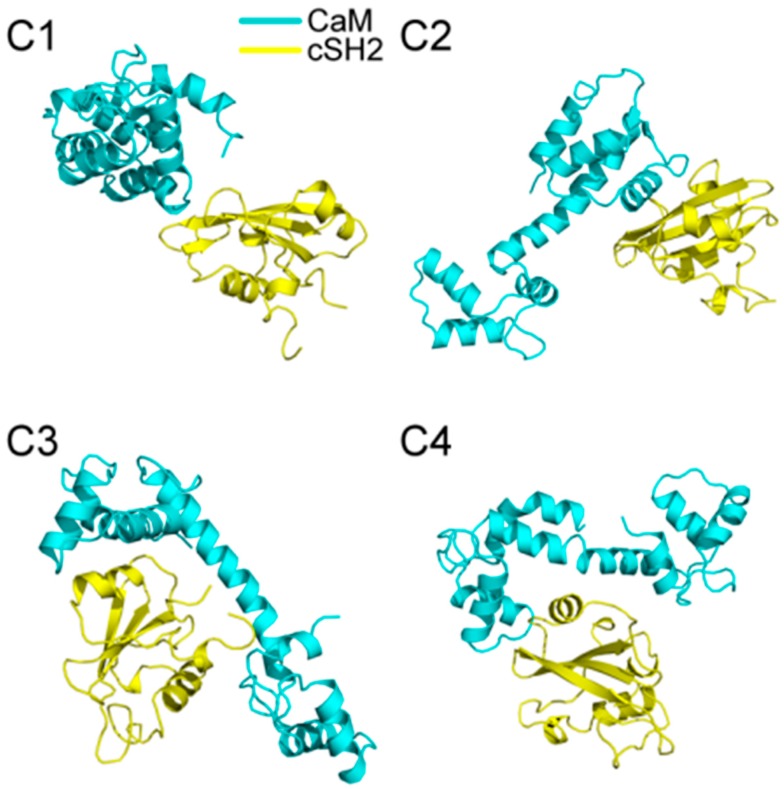
Structures of (**C1**–**C4**) complexes after 200 ns MD (molecular dynamics) simulations. Cyan structure represents CaM, and yellow for cSH2.

**Figure 2:**
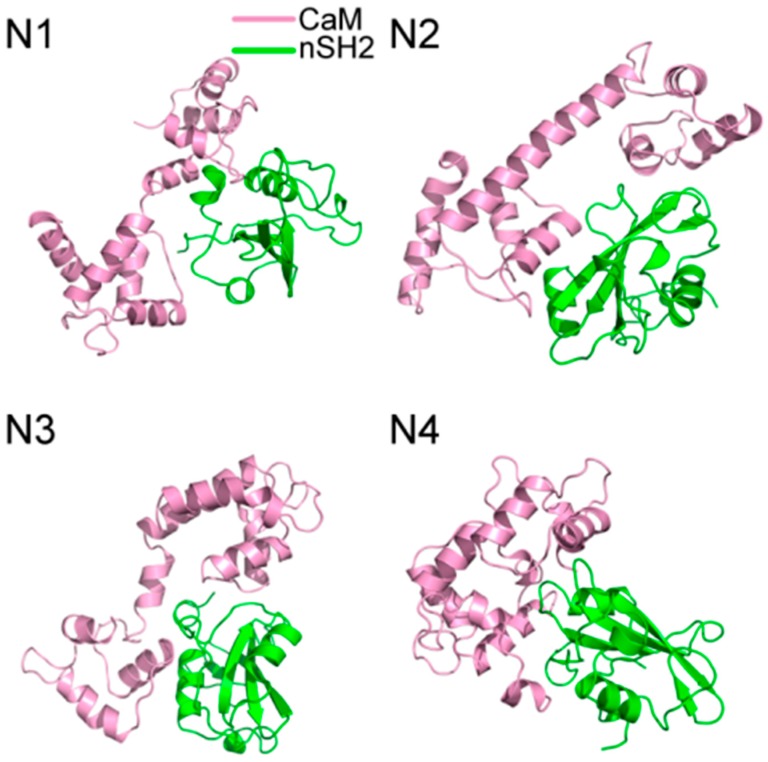
Structures of (**N1**–**N4**) complexes after 200 ns MD simulations. Pink structure represents CaM, and green for nSH2.

200 ns MD simulations were performed for the ten complex systems, including C1-C4 for CaM-cSH2, N1-N4 for CaM-nSH2, pEGFR-cSH2, and pEGFR-nSH2. We first had a brief overview of the conformation of the complexes after simulations and found that CaM can bind to the c/nSH2 domains in different topologies. For the cSH2 systems, CaM is in a collapsed conformation in C1, while in C2-C4 it adopts a stretched conformation (Figure 1). For the nSH2 systems, CaM presents a collapsed conformation in N1, N3 and N4, while in N2 it takes on a stretched conformation which enables it to wrap around nSH2 tightly (Figure 2). 

**Figure 3:**
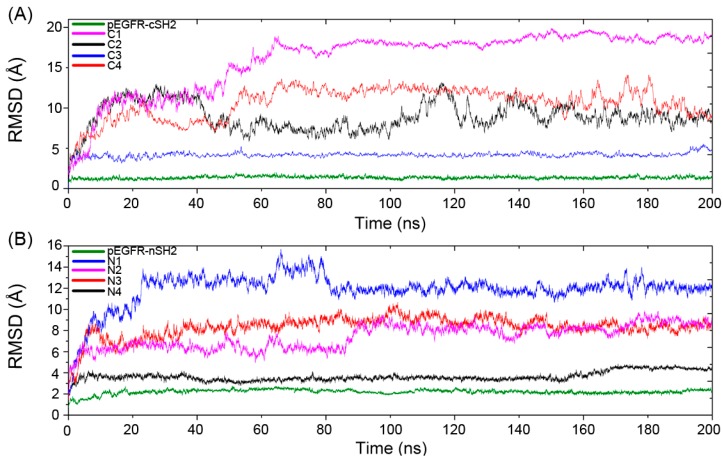
The Cα atoms root-mean-square deviation (RMSD) of eight CaM-p85α and two pEGFR-p85α systems along 200 ns MD simulations. (**A**) RMSD for CaM-cSH2 and pEGFR-cSH2 systems. (**B**) RMSD for CaM-nSH2 and pEGFR-nSH2 systems.

The Cα atoms root-mean-square deviation (RMSD) of the trajectories with respect to the original structures was calculated to reveal the dynamic conformational changes throughout the MD process. A relatively unchanged RMSD reflects the equilibrium of the simulation system and the following analysis of the trajectories only focuses on the trajectories that reach equilibrium. The equilibrium time for C1–C4 and pEGFR-cSH2 is 60, 114, 10, 60 and 30 ns, respectively, (Figure 3A) and for N1–N4 and pEGFR-nSH2, it is 20, 40, 10, 10 and 30 ns, respectively (Figure 3B).

The RMSDs in C1 and N1 are relatively large and the influence of CaM topology on RMSD contradicted with previous study. Jang et al. reported that the stretched CaM structure would yield a large RMSD while the collapsed one was related to a small value [50]. However, in our simulations, such relationship is not obvious. In C1, the collapsed CaM produces a large RMSD, while in N2 the stretched CaM produces a relatively small RMSD. Such phenomena can be explained by different interaction modes of CaM-cSH2 and CaM-nSH2. During the simulation process, CaM underwent movement or rotation around its binding site on c/nSH2, which would lead to a large RMSD for the binary complexes of CaM-cSH2 and CaM-nSH2. 

### 2.2. MM/GBSA Free Energy Analysis

To understand the energetics of the interaction between CaM or pEGFR and c/nSH2 domains, binding free energy (ΔG_binding_) of CaM or pEGFR to cSH2 or nSH2 was calculated using MM/GBSA method. As shown in Table 1, C2, C3, N2, and N3 all underwent comparable free energy changes to the pEGFR systems during binding process and the binding free energy of N4 was even lower than the pEGFR system. Insights into the compositions of the interaction energy revealed that the major contribution originated from the electrostatic term. The similar or even lower binding free energy suggests that CaM can replace pEGFR and interact with c/nSH2 domains, which supports the ternary complex hypothesis. 

To quantify the contributions of different residues to the binding free energy in details, the total energy change was decomposed into each residue by weight. Residues with free energy contributions lower than −0.3 kcal/mol were considered to be critical to the protein-protein interaction. Most of these important residues are located at the interfaces of CaM-cSH2 and CaM-nSH2 or pEGFR-cSH2 and pEGFR-nSH2, implying their pivotal roles in the protein-protein interaction. These important residues from the c/nSH2 domains reflect the effect exerted by CaM or pEGFR. We further aligned the amino acids sequences to compare the different important residues in different systems (Table 2, Figure S1). If the important residues in the CaM systems largely overlap with the ones in the pEGFR systems, it indicates that the corresponding CaM may have a pEGFR-like mode. As is shown in Table 2, C2 and N2 have the most overlapping residues with the corresponding pEGFR-p85α complex systems. In C2 there are 8 overlapping residues (Figure S1A), while in N2 there are 11 (Figure S1B). Most of these important residues are located along the interface between CaM or pEGFR and c/nSH2, suggesting that they are involved in the protein-protein interactions. More overlapping residues in the C2 and N2 also implies that the CaM in these two systems imposes a similar influence as pEGFR. Hence, it is likely that CaM can replace and mimic pEGFR’s role to interact with the c/nSH2 domains in the C2 and N2 styles, respectively. 

### 2.3. Superposition Analysis of the Candidate Structures

**Figure 4:**
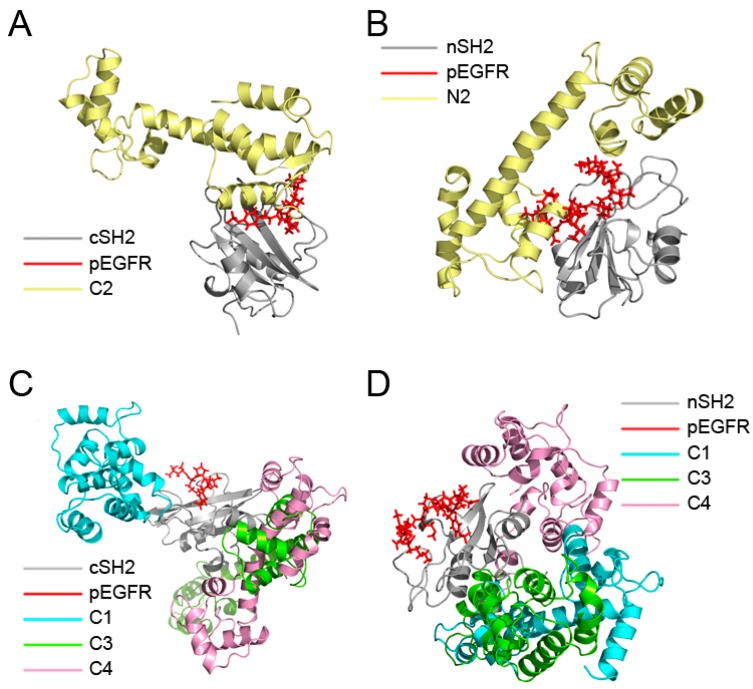
Overview of the superposition structures after 200 ns simulations. (**A**) Superposition of the structures of C2 and pEGFR-cSH2; (**B**) Superposition of the structures of N2 and pEGFR-nSH2; (**C**) Superposition of the structures of C1, C3, C4 and pEGFR-cSH2; (**D**) Superposition of the structures of N1, N3, N4 and pEGFR-nSH2. c/nSH2, pEGFR, C1/N1, C2/N2, C3/N3, and C4/N4 are colored in gray, red, cyan, pale yellow, chartreuse, and pink, respectively.

MM/GBSA analysis revealed that replacement of pEGFR by CaM is energetically favorable and the decomposition results showed that CaM in the C2 and N2 may function in a similar manner to pEGFR. To further validate these results, superposition analysis of ten complexes after simulations was carried out. C1–C4 were superposed to the pEGFR-cSH2 complex (Figure 4A,C) and N1–N4 to the pEGFR-nSH2 complex (Figure 4B,D). The results showed that CaM can bind to cSH2 or nSH2 at different sites. However, only in C2 and N2 systems the binding sites of CaM overlap with those of pEGFR (Figure 4A,B), which is in agreement with the previous energy decomposition results. 

**Figure 5:**
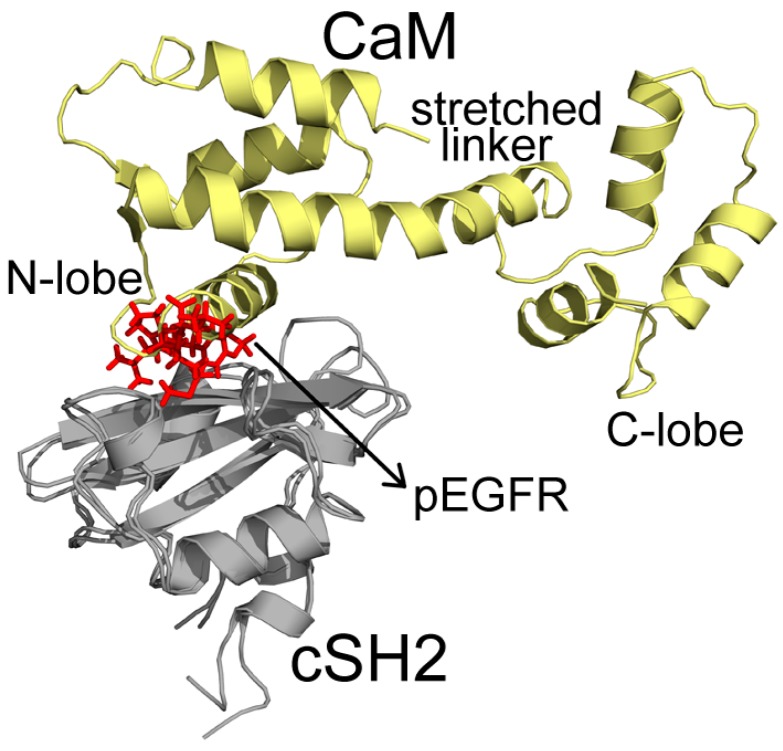
Superposition structures of C2 and pEGFR-cSH2 after 200 ns simulations. CaM, cSH2, and pEGFR are colored in pale yellow, gray, and red, respectively. Important parts of CaM structures are specified.

**Figure 6:**
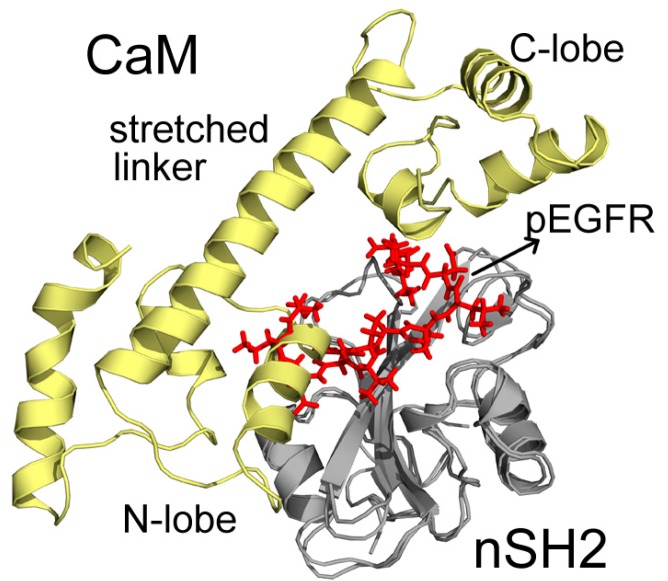
Superposition structures of N2 and pEGFR-nSH2 after 200 ns simulations. CaM, nSH2, and pEGFR are colored in pale yellow, gray, and red, respectively. Important parts of CaM structures are specified.

In C2, CaM takes on a stretched conformation, with part of its N-lobe, helix and random structure (P44-G62) inserting into the pEGFR site (Figure 5). These structures interact with the cSH2 in a similar topology to pEGFR, which can account for their similar effect. CaM binds to cSH2 near the pEGFR site and therefore can mimic the effect induced by pEGFR. In N2, CaM is in an extended conformation and it closely wraps around nSH2 and overlaps with the pEGFR binding site. The stretched linker of CaM in this system hangs across the pEGFR binding pocket and the helices in C-lobe (Y139-A148) and N-lobe (T45-D59) also interact with the pEGFR site (Figure 6). 

### 2.4. Insights into the Detailed CaM-cSH2 and CaM-nSH2 Interactions

Structural analysis showed that in C2 and N2, the binding modes of CaM are similar to the ones of pEGFR. PISA (Proteins, Interfaces, Structures and Assemblies) [51] was then employed to explore the detailed interactions of the two systems through analysis of their interfaces. 

In C2, the binding interface of CaM and cSH2 is 539.1 Å^2^, deriving from 13 residues of CaM and 21 residues of cSH2. For N2, the interface is 1217.6 Å^2^, consisting of 34 residues of CaM and 35 residues of nSH2. Similar to the free energy decomposition analysis, the interface residues on the c/nSH2 domains from the C1–C4 and N1–N4 were compared with those in pEGFR systems (Table 3, Figure S2). The interface residues in C2 and N2 significantly overlap with those in pEGFR-cSH2 and pEGFR-nSH2, which is consistent with the energy decomposition results. In addition, the interface residues detected by PISA in C2 and N2 also overlaps with those obtained from the free energy decomposition analysis.

**Figure 7:**
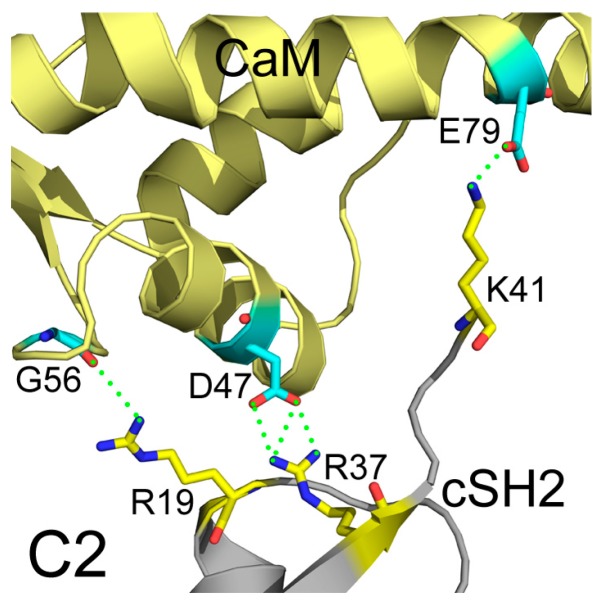
Detailed interactions between CaM and cSH2 in C2 after 200 ns simulation. Hydrogen bonds are shown as green dashed lines. CaM structure is colored in pale yellow, and cSH2 structure is in gray. CaM residues involved in hydrogen bonds formation are in cyan, and the cSH2 ones are in yellow.

**Figure 8:**
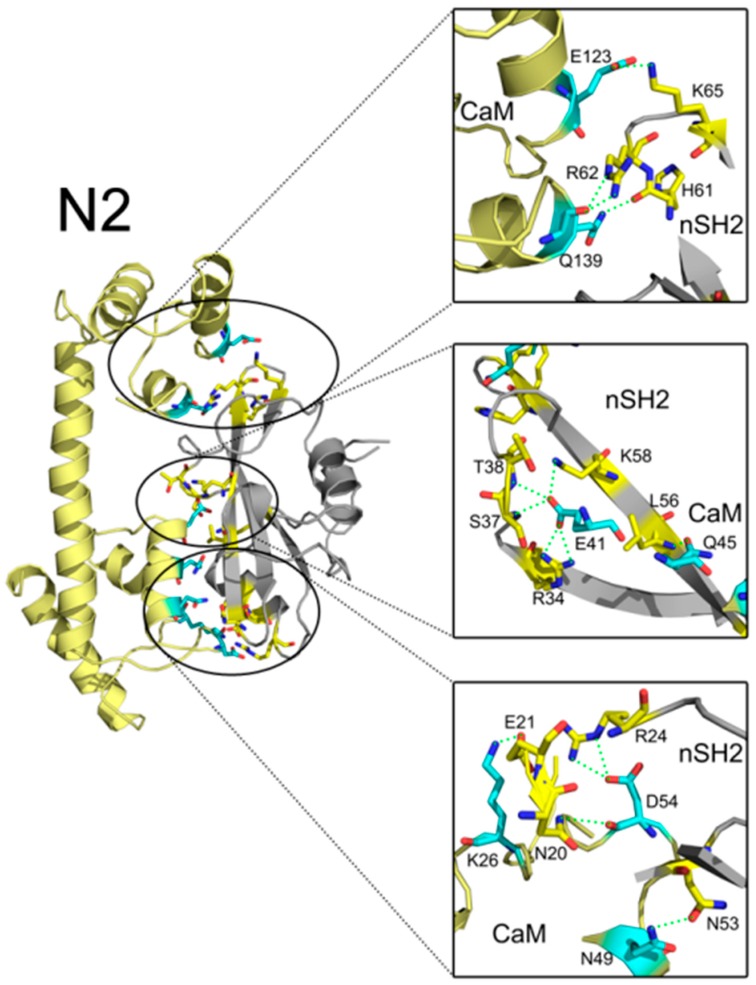
Detailed interaction between CaM and nSH2 in N2 after 200 ns simulation. Hydrogen bonds are depicted by green dashed lines. CaM structure is colored in pale yellow, and nSH2 structure is in gray. CaM residues involved in hydrogen bonds formation are in cyan, and the nSH2 ones are in yellow.

In addition to interface areas and residues, PISA also uncovered the molecular interactions along the binding interfaces. In C2, there are 5 inter-molecular hydrogen bonds and 4 salt bridges across the interface (Table S1). D47 of CaM makes salt bridges with the protonated nitrogen in the guanidine group of R37 of cSH2 and E79 of CaM forms another salt bridge with the amino group of K41 of cSH2. G56 of CaM is hydrogen-bonded to R19 of cSH2 (Figure 7). In N2, there exist 15 hydrogen bonds and 10 salt bridges between CaM and nSH2 (Figure 8, Table S2). Hotspot residues on CaM such as D54 and E41 form salt bridges with 3 neighboring residues from nSH2, revealing their critical roles within the complex. The pivotal residues involved in multiple inter-protein interactions play central parts in the binding of CaM to p85α subunit and may become potential targets for modulation of the CaM-PI3Kα protein-protein interactions. 

### 2.5. Construction of CaM-PI3Kα Complex Model

The binding modes of CaM-cSH2 and CaM-nSH2 shed light on the detailed interactions between CaM and the p85α regulatory subunit of PI3Kα. To have a view of the complete CaM-PI3Kα interaction, we constructed the full-length CaM-PI3Kα complex model. According to previous analysis, C2 and N2 are regarded as the ideal modes for CaM to interact with c/nSH2 in the p85α subunit of PI3Kα, so their protein complexes before 200 ns simulations were used to assemble our model. Crystal structure of PI3Kα (PDB ID: 4OVV) [52] was extracted from the PDB and the C2 and N2 complexes were aligned to it via structural superimposition on the c/nSH2 domains. After constructing the primary CaM-PI3Kα complex model, energy optimization of the overall systems was performed.

**Figure 9:**
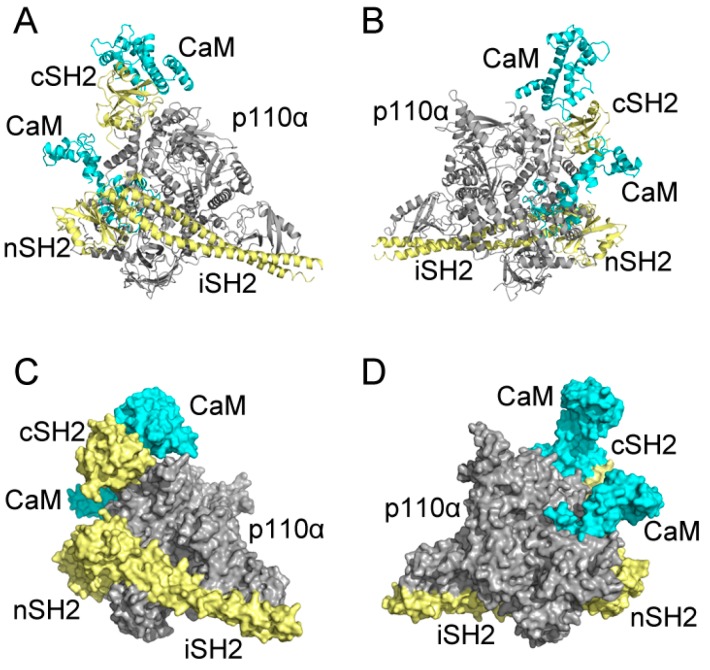
Predicted CaM-PI3Kα complex model constructed with CaM in C2 and N2 and full-length Phosphatidylinositol 3-Kinase α (PI3Kα). Complex system in cartoon mode from the front (**A**) and back (**B**); and complex system in surface mode from the front (**C**) and back (**D**). CaM is in cyan, and p110α subunit of PI3Kα is in gray, and p85α subunit, including cSH2, nSH2 and iSH2 are in pale yellow.

As shown in Figure 9, CaM takes on an extended conformation to interact with p85α in PI3Kα at the cSH2 domain. CaM places its N-lobe on the cSH2 surface and exerts a pEGFR-like effect. However, both the stretched linker and C-lobe of CaM project away, without any contact with other parts of PI3Kα. Also, in our model there exists limited space on cSH2 for CaM binding, which results in the narrow interface between CaM and cSH2. The relatively small interface may be one of the reasons for the movement of CaM throughout the interaction process and more importantly, it explains why an allosteric mechanism may be involved in the activation of PI3Kα by CaM through cSH2 [18]. Given the narrow interface between CaM and cSH2, direct protein-protein contact may not be able to exert effects strong enough and hence, allosteric signaling may play a role in the activation of PI3Kα by CaM through cSH2. As for the nSH2 domain, previous comparison of N2 structures before and after simulation shows that CaM moved along the nSH2 interface during simulation and this observation can find its origin in our assembly. In our model, the structures near nSH2 are relatively flexible and the inserting CaM does not have much conformational hindrance around its binding site. Hence, it is very likely that when interacting with full-length PI3Kα CaM will also move across the nSH2 interface, inserting towards the inner part of PI3Kα and impose its activating effect. Moreover, nSH2 situates near iSH2 domain (part of p85α subunit that connects nSH2 to cSH2) and p110α subunit of PI3Kα and the CaM binding site locates adjacent to the interface between p110α and nSH2. Since there are relatively fewer conformational constraints around the interfaces, these observations support our simulation results, in which CaM gradually “crawls” across nSH2 and inserts into the whole protein assembly to exert its influence. 

## 3. Discussion

CaM and PI3Kα are known to regulate several cellular events [22,23,24,53]. In addition, there exists crosstalk between them and their related intracellular pathways. Under normal circumstances, PI3Kα is activated by pEGFR and K-Ras4B together. pEGFR binds to c/nSH2 domains of the p85α regulatory subunit of PI3Kα, which can relieve the inhibitory effect of p85α on its p110α catalytic subunit. After that, K-Ras4B binds to PI3Kα and allosterically stimulates the production of PIP_3_ [47,48]. Previously, CaM has been reported to be able to activate PI3Kα through p85α subunit independent of K-Ras4B [45,46], and recent research suggests that CaM can replace pEGFR and activate PI3Kα aberrantly [47,48,49]. However, details behind this interaction still remain elusive. Unraveling the mechanisms underlying CaM-PI3Kα crosstalk through which CaM abnormally replaces pEGFR and activates PI3Kα is expected to contribute to future research and clinical application. 

Here, by MD simulations we found that CaM can bind to c/nSH2 domains with comparable reaction free energy to pEGFR, implying that the replacement of pEGFR by CaM is feasible. Through structure superposition and interface analysis, C2 complex and N2 complex were predicted to be the ideal models for CaM-cSH2 and CaM-nSH2 interactions, in which CaM adopts similar acting modes to pEGFR. The details of the interactions in these two systems including hydrogen bonds and salt bridges were unmasked, and they are the origin of the tight binding of CaM and its ability to induce the pEGFR-like effect. The hotspot residues for CaM-cSH2 and CaM-nSH2 interactions were identified, which will be instructive to future related study. Moreover, based on these findings we constructed a CaM-PI3Kα complex assembly, which provides guidance for further exploration. Similarly, Zhang et al. also explored the crosstalk between CaM and PI3K lately, but they focus on phosphorylated CaM at Y99 [45]. Both of our studies produce similar results supporting each other, in which the CaM takes on similar topologies to interact with PI3Kα and exerts the activating effect. Our research supplies in-depth insights into the CaM-PI3Kα crosstalk, and will be of great significance to future relevant research and application. 

Recently, we put forward a novel theory about the CaM-PI3Kα interaction where K-Ras4B is also involved and they form a ternary complex together, especially in carcinogenesis [47,48,49]. Details of this hypothesis remain unresolved and this gap can be partly filled by our research, which provides underlying mechanisms of the CaM-cSH2 and CaM-nSH2 interactions. Moreover, our findings not only supply solid support for this hypothesis, but also provide instructions for therapies towards the K-Ras4B-driven adenocarcinoma regulated by CaM and PI3Kα. Modulations of protein-protein interaction (PPI) have already become a promising idea in drug development [13,47,54,55], and PPIs inhibitors as well as related drugs can be designed based on the interaction modes and hotspot residues uncovered here. Very recently, we have found some potential allosteric targets on Ras [47,56,57,58,59]. Combining our previous findings and the discovery here, we will be able to target the ternary complex, which can prevent the oncogenesis of the K-Ras4B-driven adenocarcinoma. Hence, our study here provides crucial theoretical basis for further research on CaM and PI3Kα as well as sheds light on future clinical adenocarcinoma therapy and drug development. 

## 4. Materials and Methods

### 4.1. Construction of Simulation Systems

For the pEGFR-nSH2 system, the crystal structure of nSH2 domain of the p85α regulatory subunit of PI3Kα and platelet-derived growth factor receptor (PDGFR) peptide (PDB ID: 2IUI), which can mimic the domain of pEGFR that interacts with the nSH2 domain of PI3Kα [60], was extracted from the RCSB Protein Data Bank (PDB). For the PDGFR peptide and PI3Kα cSH2 system, 1H9O was chosen from the PDB [61]. 

As for the CaM-cSH2 and CaM-nSH2 systems, no structures are available currently. PRISM [62,63,64] was employed to carry out the inter-protein interaction prediction to set up the complex systems for further MD simulations. PRISM compared patches along the surfaces of the two interacting proteins, and if they had similar structural features to two complementary sides of a template interface, and the “hot spot” residues on both the proteins and templates were evolutionarily conserved, these two proteins would be regarded to be likely to interact with each other. Such comparison not only considered the rigid structure similarity, but also took the backbone and side-chain flexibility into account [63,64]. Finally, such kind of interaction would be graded according to the binding free energy and the different contribution terms of it, including desolvation energy, van der Waals interactions, partial electrostatics and so on [65,66]. We first downloaded all the available structures of CaM, cSH2, and nSH2 from PDB, and PRISM prediction was then carried out based on these structural data. CaM was docked to cSH2 and nSH2 respectively, and the top four complexes with highest PRISM scores in these two systems were chosen, and would be further simulated and analyzed [67,68,69]. These eight complexes structures were denoted as the C1 to C4 and N1 to N4 for the cSH2 and nSH2 systems, respectively. The compositions of these systems are shown in Table 4 and MD simulations were then performed. 

### 4.2. MD Simulations

MD simulations for the ten systems described above (C1-C4, N1-N4, pEGFR-cSH2, and pEGFR-nSH2) were carried out using Amber 14 (San Francisco, CA, USA) [70]. The modified ff03 force field [71] was applied to the calculation of the force field parameters of the complex systems. All the complexes above first underwent solvation using the TIP3P water model, and Na^+^ counterions were added to neutralize the whole systems. The energy of the systems was minimized after the preparation process. The energy minimization could be divided into two parts, and in the first part, the proteins structures were held in place, and 5000 steps of minimization cycles were carried out to minimize the energy of the water molecules and the counter ions. After that, complexes were relaxed and the second round of minimization without restriction followed. Once the system energy had been minimized, they were heated from 0 to 300 K within 300 ps, under 10 kcal/(mol × Å^2^) positional restraint in a canonical ensemble (NVT), and then equilibration of the system was carried out at the target temperature, 300 K, under the same conditions for 700 ps. Finally, all the complexes underwent 200 ns MD simulations in isothermal and isobaric ensemble with periodic boundary conditions. Long-range electrostatic interactions were solved with the help of the particle mesh Ewald method, and a 10 Å cut-off was employed to deal with the short-range electrostatics and van der Waals interactions. Within systems, all hydrogen-involved covalent bonds were restricted by the SHAKE method, and the final trajectories were written out every 20 ps. 

### 4.3. Molecular Mechanics Generalized Born Surface Area Calculations

In Amber 14 [70], plugin MMPBSA.py was used to carry out the Molecular Mechanics Generalized Born Surface Area (MM/GBSA) calculations. Free energy was calculated for the whole complex, receptor (CaM), and ligand (nSH2, cSH2 or pEGFR) correspondingly. The free energy of the binding reaction came as a result of the following Equation (1):

ΔG = G_complex_ − G_receptor_ − G_ligand_ (1)

In Equation (1), free energy (G) was calculated according to Equation (2):

ΔG = ΔE_gas_ + ΔG_solvation_ − TΔS (2)

In which, ΔE_gas_ represented the gas phase molecular mechanical energy, ΔG_solvation_ stood for the solvation free energy, and −TΔS for the entropy term. ΔE_gas_ could be further divided into three parts, as is shown in Equation (3), and it equaled to the sum of the van der Waals energy (ΔE_vdW_), electrostatic energy (ΔE_ele_) and gas phase internal energy (ΔE_int_):

ΔE_gas_ = ΔE_vdW_ + ΔE_ele_ + ΔE_int_ (3)

Continuum solvent methods were employed to calculate ΔG_solvation_, which consisted of polar contribution (ΔG_PB/_) and non-polar contribution (ΔG_nonpolar_), given as Equation (4):

ΔG_solvation_ = ΔG_PB_ + ΔG_nonpolar_ (4)

Calculation of the electrostatic solvation energy was based on the finite difference PB model, in which the solute and water dielectric constants were chosen to be 1 and 80 respectively. Equation (5) was used to work out the non-polar contribution (ΔG_nonpolar_) to the solvation free energy (ΔG_solvation_):

ΔG_solvation_ = γSASA + b (5)

In Equation (5), solvent-accessible surface-area was abbreviated as *SASA*, solvation parameter γ equaled 0.00542 kcal (mol^−1^∙Å^−2^) and the other solvation parameter, b, was 0.92 kcal/mol.

The conformation entropy (−TΔS) was omitted in our calculations. This term is usually estimated with quasi harmonic analysis of the simulation trajectories using normal mode analysis, which demands a lot of computational time. Since in our study, we only focused on the relative ordering of binding free energy, and throughout the interaction process, the interaction modes and the overall root-mean-square deviation (RMSD) of the Cα atoms with respect to the original structures during simulations did not change significantly. Taking all these into consideration, we left out the conformation entropy term in our free energy calculation. The overall free energy difference ΔG was distributed to every residue within the system by weight with the help of the MM/GBSA method in Amber 14.

## 5. Conclusions

CaM-PI3Kα crosstalk is a common and central process within series of cellular activities and human physiological or pathological conditions. Here in our study, using MD simulations and molecular modeling, we explored into the detailed interaction modes between CaM and the cSH2 and nSH2 domains of the p85α regulatory subunit in PI3Kα. We first demonstrated that CaM can interact with these two domains from an energetic view and by structural superposition we found out the potential ideal binding poses for CaM to interact with cSH2 and nSH2. Detailed inter-molecular interactions such as salt bridges and hydrogen bonds were revealed and the corresponding hotspot residues for protein-protein interactions were also unmasked. With these finding, we also assembled the full-length CaM-PI3Kα complex, which is the first report for this interaction system. Our study not only provides solid theoretical basis for relevant research but also shed light on related targeting drug discovery. 

## Figures and Tables

**Table 1:** Free energy analysis (kcal/mol) for the CaM/pEGFR-p85α interactions ^a^.

**Systems**	**C1**	**C2**	**C3**	**C4**	**pEGFR-cSH2**
DE_ele_	−487.24 (72.84)	−454.04 (56.60)	−481.67 (72.71)	−648.54 (67.12)	−272.97 (176.16)
DE_vdW_	−28.32 (8.80)	−52.71 (5.67)	−104.44 (9.22)	−44.78 (8.30)	−26.16 (18.75)
DG_nonpolar_	−4.84 (1.46)	−7.08 (0.98)	−14.41 (1.35)	−6.74 (1.05)	−4.72 (3.31)
DG_polar_	498.13 (73.12)	476.20 (53.19)	546.28 (67.34)	677.47 (67.24)	264.58 (170.14)
DG_binding_	−17.43 (11.54)	−30.56 (10.19)	−39.83 (10.46)	−15.84 (7.87)	−34.56 (24.51)
**Systems**	**N1**	**N2**	**N3**	**N4**	**pEGFR-nSH2**
DE_ele_	−327.90 (66.64)	−696.61 (78.38)	−445.38 (102.15)	−750.52 (89.50)	−420.68 (242.11)
DE_vdW_	−73.53 (9.76)	−78.07 (13.17)	−101.85 (11.04)	−132.88 (10.91)	−40.35 (23.74)
DG_nonpolar_	−11.06 (1.25)	−12.18 (1.79)	−14.69 (1.73)	−20.70 (1.41)	−6.71 (3.88)
DG_polar_	370.08 (62.20)	718.05 (75.83)	484.00 (98.17)	787.72 (86.24)	395.50 (226.89)
DG_binding_	−31.35 (10.49)	−56.62 (10.88)	−63.23 (12.84)	−95.68 (11.78)	−65.53 (38.63)

^a^ Numbers in the parentheses present the standard deviations. The MM/GBSA binding free energy (DG_binding_ = DE_vdW_ + DE_ele_ + DG_nonpolar_ + DG_polar_).

**Table 2:** Comparison of the important residues for CaM/pEGFR-p85α interactions.

**System**	**C1**	C2 *	**C3**	**C4**
Overlap residues	5	8	4	3
**System**	**N1**	N2 *	**N3**	**N4**
Overlap residues	5	11	5	5

* Systems with asterisks are the ones with most overlapping residues important for interactions.

**Table 3:** Comparison of the interface residues of CaM/pEGFR-p85α interactions.

**System**	**C1**	C2 *	**C3**	**C4**
Overlap residues	2	14	0	0
**System**	**N1**	N2 *	**N3**	**N4**
Overlap residues	0	15	2	5

* Systems with asterisks are the ones with most overlapping interface residues.

**Table 4:** Compositions of the predicted CaM-cSH2 and CaM-nSH2 complexes by PRISM.

**System**	**C1**	**C2**	**C3**	**C4**
CaM	1CDL *	1CLL	1CLL	1CLL
cSH2	1H9O	1H9O	1H9O	1H9O
**System**	**N1**	**N2**	**N3**	**N4**
CaM	2BE6	1S26	2BE6	2BE6
nSH2	2IUG	2IUH	2IUG	2IUH

* The PDB ID of the protein structure.

## Supplementary Materials

Supplementary materials can be found at www.mdpi.com/1422-0067/19/1/151/s1.
