# Supplementary material for: Computational Insights into the Interactions between Calmodulin and the c/nSH2 Domains of p85α Regulatory Subunit of PI3Kα: Implication for PI3Kα Activation by Calmodulin
Source: Int J Mol Sci. 2018 Jan 4;19(1):151. doi: 10.3390/ijms19010151 (PMC5796100; doi:10.3390/ijms19010151)
Supplement: Supplementary file 1 [file ijms-19-00151-s001.pdf]

Supplementary

A

10 20 30 40 50 60

pEGFR-cSH2 GSPIPHHDEK TWNVGSSNRN KAENLLRGKR DGTFLVRESS KQGCYACSVV VDGEVKHCVI

C1 GSPIPHHDEK TWNVGSSNRN KAENLLRGKR DGTFLVRESS KQGCYACSVV VDGEVKHCVI

C2 GSPIPHHDEK TWNVGSSNRN KAENLLRGKR DGTFLVRESS KQGCYACSVV VDGEVKHCVI

C3 GSPIPHHDEK TWNVGSSNRN KAENLLRGKR DGTFLVRESS KQGCYACSVV VDGEVKHCVI

C4 GSPIPHHDEK TWNVGSSNRN KAENLLRGKR DGTFLVRESS KQGCYACSVV VDGEVKHCVI

70 80 90 100 109

pEGFR-cSH2 NKTATGYGFA EPYNLYSSLK ELVLHYQHTS LVQHNDSLNV TLAYPVYAQ

C1 NKTATGYGFA EPYNLYSSLK ELVLHYQHTS LVQHNDSLNV TLAYPVYAQ

C2 NKTATGYGFA EPYNLYSSLK ELVLHYQHTS LVQHNDSLNV TLAYPVYAQ

C3 NKTATGYGFA EPYNLYSSLK ELVLHYQHTS LVQHNDSLNV TLAYPVYAQ

C4 NKTATGYGFA EPYNLYSSLK ELVLHYQHTS LVQHNDSLNV TLAYPVYAQ

PI3K p85α cSH2 domain

B

10 20 30 40 50 60

pEGFR-nSH2 ---MSLQNAEW YWGDISREEV NEKLRDTADG TFLVRDASTK MHGDYTLTLR KGGNNKLIKI

N1 NNMSLQNAEW YWGDISREEV NEKLRDTADG TFLVRDASTK MHGDYTLTLR KGGNNKLIKI

N2 --NMSLQNAEW YWGDISREEV NEKLRDTADG TFLVRDASTK MHGDYTLTLR KGGNNKLIKI

N3 NNMSLQNAEW YWGDISREEV NEKLRDTADG TFLVRDASTK MHGDYTLTLR KGGNNKLIKI

N4 --NMSLQNAEW YWGDISREEV NEKLRDTADG TFLVRDASTK MHGDYTLTLR KGGNNKLIKI

70 80 90 100 110

pEGFR-nSH2 FHRDGKYGFS DPLTFSSVVE LINHYRNESL AQYNPKLDVK LLYPVSKYQ 107

N1 FHRDGKYGFS DPLTFSSVVE LINHYRNESL AQYNPKLDVK LLYPVSKYQQ 110

N2 FHRDGKYGFS DPLTFSSVVE LINHYRNESL AQYNPKLDVK LLYPVSKYQQ DQVVKE 115

N3 FHRDGKYGFS DPLTFSSVVE LINHYRNESL AQYNPKLDVK LLYPVSKYQQ 110

N4 FHRDGKYGFS DPLTFSSVVE LINHYRNESL AQYNPKLDVK LLYPVSKYQQ DQVVKE 115

PI3K p85α nSH2 domain

Figure S1. Sequence alignment of C1-C4 and N1-N4 to pEGFR-cSH2 and pEGFR-nSH2 to find the overlap of the residues important for CaM-p85α and pEGFR-p85α interactions. Important residues are in red.

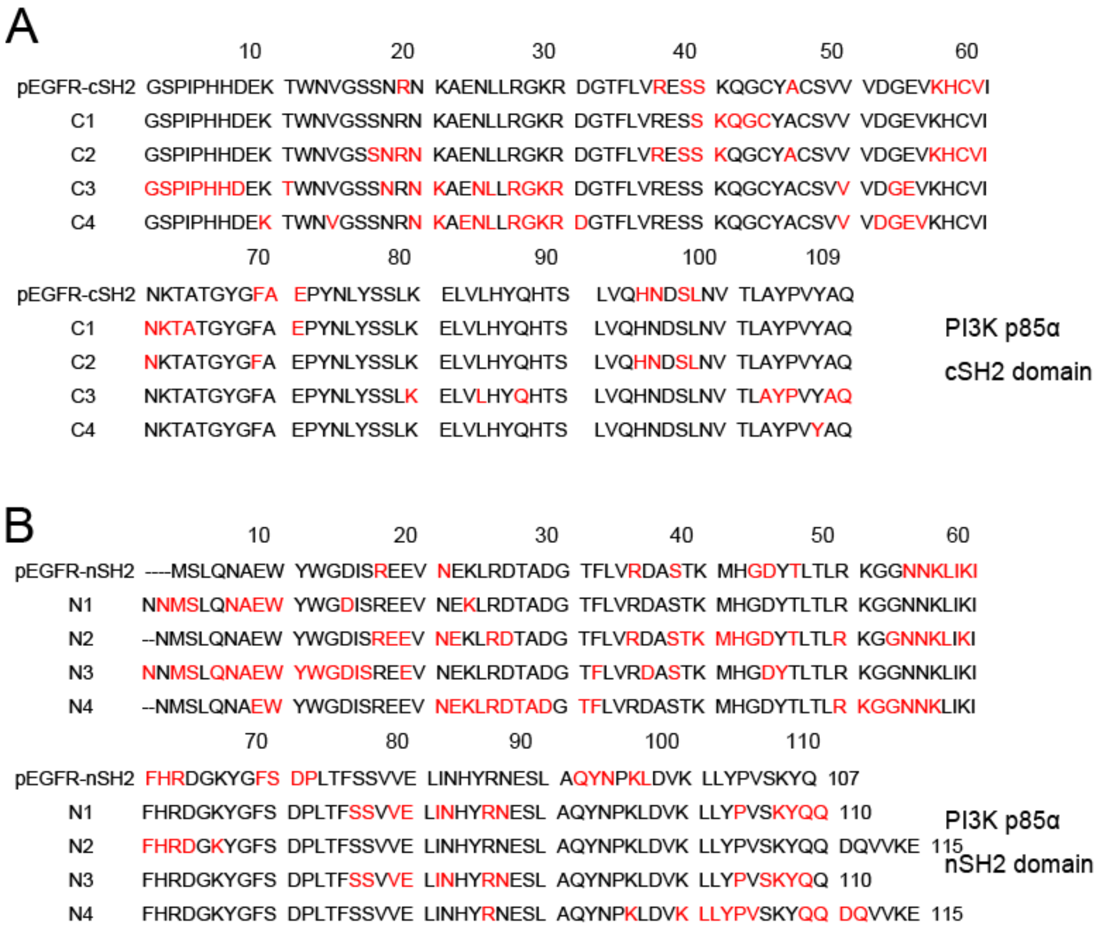

Figure S2. Sequence alignment of C1-C4 and N1-N4 to pEGFR-cSH2 and pEGFR-nSH2 to find the overlap of the interface residues (red) within CaM-p85α and pEGFR-p85α interactions.

Table S1. Summary of the hydrogen bonds and salt bridges formed between CaM and cSH2 in the C2

Number	H Bonds	Distance (Å)
Hb1	CaM-D47-OD1···cSH2-R37-NH1	3.20
Hb2	CaM-D47-OD1···cSH2-R37-NH2	2.83
Hb3	CaM-D47-OD2···cSH2-R37-NH1	2.98
Hb4	CaM-G56-O···cSH2-R19-NH1	3.66
Hb5	CaM-E79-OE1···cSH2-K41-HZ3	1.94
Number	Salt Bridge	Distance (Å)
Sb1	CaM-D47-OD1···cSH2-R37-NH1	3.20
Sb2	CaM-D47-OD1···cSH2-R37-NH2	2.83
Sb3	CaM-D47-OD2···cSH2-R37-NH1	2.98
Sb4	CaM-E79-OE1···cSH2-K41-NZ	2.87

Table S2. Summary of the hydrogen bonds formed between CaM and nSH2 in the N2

Number	H Bonds	Distance (Å)
Hb1	CaM-K26-HZ2···nSH2-E21-OE2	1.78
Hb2	CaM-N49-HD21···nSH2-N53-OD1	1.81
Hb3	CaM-Q139-HE22···nSH2-H61-O	2.24
Hb4	CaM-D54-O···nSH2-N20-HD21	2.35
Hb5	CaM-D54-OD1···nSH2-R24-HE	2.05
Hb6	CaM-D54-OD1···nSH2-R24-HH11	1.81
Hb7	CaM-E41-OE1···nSH2-R34-HH12	1.96
Hb8	CaM-E41-OE1···nSH2-R34-HH22	1.85
Hb9	CaM-E41-OE2···nSH2-S37-H	1.96
Hb10	CaM-E41-OE2···nSH2-T38-H	2.08
Hb11	CaM-Q45-OE1···nSH2-L56-H	2.28
Hb12	CaM-E41-OE2···nSH2-K58-HZ2	1.99
Hb13	CaM-Q139-O···nSH2-R62-HE	1.68
Hb14	CaM-Q139-O···nSH2-R62-HH11	2.23
Hb15	CaM-E123-OE2···nSH2-K65-HZ1	1.96
Number	Salt Bridge	Distance (Å)
Sb1	CaM-K26-NZ···nSH2-E21-OE1	3.57
Sb2	CaM-K26-NZ···nSH2-E21-OE2	2.76
Sb3	CaM-D54-OD1···nSH2-R24-NE	2.97
Sb4	CaM-D54-OD2···nSH2-R24-NE	3.37
Sb5	CaM-D54-OD1···nSH2-R24-NH1	2.76
Sb6	CaM-E41-OE1···nSH2-R34-NH1	2.85
Sb7	CaM-E41-OE1···nSH2-R34-NH2	2.71
Sb8	CaM-E41-OE2···nSH2-K58-NZ	2.90
Sb9	CaM-E123-OE2···nSH2-K65-NZ	2.83
Sb10	CaM-E123-OE1···nSH2-K65-NZ	3.33
